# Costs of Rapid HIV Screening in an Urban Emergency Department and a Nearby County Jail in the Southeastern United States

**DOI:** 10.1371/journal.pone.0128408

**Published:** 2015-06-08

**Authors:** Anne C. Spaulding, Robin J. MacGowan, Brittney Copeland, Ram K. Shrestha, Chava J. Bowden, Min J. Kim, Andrew Margolis, Genetha Mustaafaa, Laurie C. Reid, Katherine L. Heilpern, Bijal B. Shah

**Affiliations:** 1 Department of Epidemiology, Rollins School of Public Health, Emory University, Atlanta, GA, United States of America; 2 Centers for Disease Control and Prevention, Atlanta, GA, United States of America; 3 Department of Emergency Medicine, Emory School of Medicine, Atlanta, GA, United States of America; Old Dominion University, UNITED STATES

## Abstract

Emergency departments and jails provide medical services to persons at risk for HIV infection and are recommended venues for HIV screening. Our main objective in this study was to analyze the cost per new HIV diagnosis associated with the HIV screening program in these two venues. The emergency department’s parallel testing program was conducted at Grady Memorial Hospital in Atlanta, Georgia starting in 2008; the jail’s integrated testing program began at the Fulton County (GA) Jail in 2011. The two sites, four miles apart from one another, employed the same rapid HIV test. Ascertainment that cases were new differed by site; only the jail systematically checked identities against health department HIV registries. The program in the emergency department used dedicated HIV test counselors and made 242 diagnoses over a 40-month period at a cost of $2,981 per diagnosis. The jail program used staff nurses, and found 41 new HIV cases over 10.5 months at a cost of $6,688 per new diagnosis. Differences in methods for ascertainment of new diagnoses, previously undiagnosed HIV sero-positivity, and methodologies used for assessing program costs prevent concluding that one program was more economical than the other. Nonetheless, our findings show that testing in both venues yielded many new diagnoses, with the costs within the range reported in the literature.

## Introduction

In 2010, the White House released the first comprehensive National HIV/AIDS Strategy. [1] Since the publication of this Strategy, data released by the Centers for Disease Control and Prevention show that there are significant gaps along the continuum of HIV care—the sequential stages of care from diagnosis to viral suppression. [2] Nearly one in six of the estimated 1.1 million people living with HIV in the United States are unaware of their infection and an estimated 48,000 new infections occur each year.[3] Implementing HIV screening in a cost efficient manner in venues with large numbers of persons who may not otherwise be tested is an important step in reducing the number of persons unaware of their infection and improving linkage to subsequent medical care.

Emergency departments (EDs) and jails provide medical services to persons at risk for HIV infection and are recommended venues for HIV screening,[4,5] but typically less than 1% of persons entering either venue are tested for HIV.[6,7] Previous studies have highlighted the cost-effectiveness of HIV testing in EDs and jails and the importance of identifying newly diagnosed HIV cases.[8,9] Despite the CDC guidelines for routine HIV screening in healthcare settings [5] and CDC guidance to correctional facilities on incorporating HIV testing into routine medical services,[4] surveys have shown fewer than a quarter of EDs in the U.S. offer systematic HIV testing programs, and the largest jails in most communities where HIV prevalence is high do not routinely provide HIV screening during the intake medical evaluation.[6,7]

Safety-net hospitals and jails located in the inner-cities often manage similar populations and provide health care to persons experiencing health disparities, including those who are underinsured, low socio-economic status, and racial and ethnic minorities. Studies conducted in Georgia have shown neighborhoods disproportionately affected by poverty have a higher than average percentage of residents with a history of incarceration.[10] Not only are populations of the two institutions shared, but the processes by which people are evaluated for medical services in the venues are similar. Nationally, an ED in a public, safety-net hospital has a median length of stay of 5.0–7.5 hours.[11] A jail is a short-term correctional facility, operated by a local municipality or county, where the median length of stay is 2 to 5 days.[12,13] In both venues, rapid throughput of entrants is an important goal. Staff may express concerns that adding routine HIV screening could overburden the organization’s ability to meet that goal. Nonetheless, evidence has shown that incorporating HIV testing into the operations of both EDs and jails is feasible.[14,15] Cost data on ED and jail-based HIV screening programs are necessary for program administrators considering providing routine HIV screening in these settings.

Grady Memorial Hospital (GMH) and the Fulton County Jail (FCJ) are located approximately 4.5 miles apart in the downtown area of Atlanta, Georgia. At the end of 2010, 18.2% of county residents were living below the poverty level and 1.3% of Fulton County residents were infected with HIV.[16,17] Previous studies have documented that HIV screening at both GMH and the FCJ venues identify a high number of HIV infections, including infections among men who have sex with men and racial minorities.[18–20] This paper describes the cost of HIV screening in the Grady Memorial Hospital Emergency Department (GMHED), which sees primarily adult patients, and the Fulton County Jail, and reports the cost per newly diagnosed case of HIV infection detected in each of the two screening programs.

## Methods

In June 2008, the GMHED initiated a rapid HIV screening program utilizing a parallel staffing model whereby HIV testing staff offer testing separate from the regular ED medical services.[18,19] The program continued through December 2011, for a total of 40 months. The program was staffed by three part-time counselors, one part-time database manager, one full-time program coordinator and an ED physician with funded time (0.15 FTE) for program supervision. Patients were identified by the HIV testing staff as ineligible for HIV screening if they were less than 18 years of age, known to have HIV/AIDS, non-English speaking, incarcerated, medically unstable or unable to decline testing. The HIV counselors approached eligible patients from 10:00am to 10:00pm Monday through Friday in either the ED waiting room or in individual examination rooms. The trained HIV counselors reviewed the triage list and approached eligible patients in consecutive order, and explained that rapid HIV screening was part of routine medical care. In accordance with hospital requirements, patients were given the opportunity to decline the HIV screening test and were asked to sign a form indicating their decision to accept or decline. Patients accepting HIV screening received pre-test prevention counseling. GMHED’s HIV testing data were collected prospectively for quality assurance and analyzed retrospectively for research purposes. The retrospective analysis was approved by Emory University’s Institutional Review Board (IRB) and Grady Memorial Hospital’s Research Oversight Committee.

In January 2011, nursing staff at the FCJ began integrated routine rapid HIV screening into the medical intake process, which occurs immediately after booking. Data were analyzed through December 2011 (excluding 6 weeks midsummer 2011, when the contractor providing medical services for FCJ changed). Nurses provided HIV screening 24 hours a day, 7 days per week during medical intake evaluations. Prior to intake, nursing staff obtained written consent from the detainees for general medical care, including HIV screening. Nurses informed the detainee-patient that voluntary HIV screening was a routine screening test conducted during the intake medical evaluation and the patient had the right to decline the test. The extensiveness of HIV prevention counseling provided was based on the clinical judgment of the nurse. HIV risk information was obtained for all persons with a reactive HIV test result. All detainees with a reactive rapid test result were referred for additional HIV medical evaluation. Additionally, an HIV educator provided on-going HIV training to the nursing staff and visited newly diagnosed persons within one day of the preliminary diagnosis to supplement counseling provided by the nurses. HIV-positive patients or those determined by nursing staff to be mentally incompetent were not screened for HIV. The protocol for this program was reviewed by Emory University’s IRB and determined that it was public health practice rather than human subjects’ research.

### HIV Testing Algorithm

Both the GMHED and FCJ programs used OraQuick ADVANCE Rapid HIV-1/2 Antibody Test (OraSure Technologies, Bethlehem PA) to detect antibodies to HIV-1 and/or HIV-2 in an oral fluid specimen. This second-generation, CLIA-waived rapid HIV test provided results in 20 minutes.[21] All patients testing preliminarily positive were asked to provide a venous blood specimen for confirmatory testing. Only patients with positive Western blot tests are considered confirmed as HIV-positive in this report. Persons with preliminary positive tests with negative Western blots were reclassified as HIV-negative and were not included in the subsequent counts of HIV-positive persons.

### New HIV Diagnosis

The GMHED did not implement a standardized procedure for determining if patients were newly reported to the health department’s surveillance registry. However, an HIV/AIDS case report form was completed on all patients testing Western blot positive and submitted to both the State of Georgia’s Department of Public Health Epidemiology Section and the Fulton County Department of Health and Wellness. Fulton County surveillance staff routinely, but not systematically, informed GMHED staff when a patient’s HIV infection had been previously reported to the registry. In addition, during post-test counseling the program staff queried a patient testing positive once more regarding previous HIV test results. The patient’s GMH chart was also extensively reviewed for evidence of a previous HIV diagnosis.

In contrast, the FCJ staff submitted names of each detainee testing Western blot positive to the HIV surveillance registries of the Fulton County Department of Health and Wellness and the State of Georgia’s Department of Public Health, and the FCJ staff were systematically informed whether the diagnosis was new. In addition, the charts of detainees newly diagnosed with HIV were reviewed to determine if they disclosed to a health provider a prior HIV diagnosis, such as one made in another state and not captured by Georgia HIV surveillance registries.

### Cost Analysis

Expenses associated with rapid HIV test kits, supplies, and labor, were recorded for each program. For both HIV screening programs, costs were calculated from the service provider’s perspective. Costs related to time and productivity of the patients, facility space, and any durable items, (e.g., computer, printer, refrigerator, etc.) were excluded. Recurrent costs of test kits (including cost of donated test kits), supplies, and labor were included. Costs were analyzed at the GMHED based on the program expenditure, which involves looking at the actual amount of funding that was disbursed or recommended to determine program cost.[22] The GMHED’s labor costs were provided by program staff from program expenditure reports.[22] For FCJ, data were analyzed via microcosting and staff allocation methods, which involve looking at the proportion of each staff person’s time that was spent working on the program. [22] The FCJ’s labor costs for counseling and testing were determined by a time-motion study of HIV testing by nurses one shift a week for three weeks, conducted during November 2011, and the remainder of the labor costs were based on staff allocation. All costs were reported in 2011 US dollars.

### Data Management

GMHED staff completed a paper-based CDC HIV testing form on each patient tested for HIV. The data from the forms were subsequently entered into a secure SPSS database (IBM, Armonk, NY) by program staff. The FCJ program staff maintained an Excel database (Microsoft database, Redmond WA) with each tested patient’s demographic information, as well as a paper-based nursing log for each tested patient. Emory staff entered these data manually into REDCap, a secure, web-based, electronic data capture tool.[23]

## Results

The GHMED had approximately 360,000 patient visits during the 40-month study period. 19,388 eligible patients were approached: no risk behavior assessment or determination of signs or symptoms was conducted before offering HIV screening. Of those that were offered the test, 15,510 (80.0%) were tested (7,562 men, 7,946 women and 2 male-to-female transgender persons). Two hundred and sixty-eight oral fluid tests were preliminarily positive. Project staff confirmed that 26 persons had been previously diagnosed. The remaining 242 (90.3%) were confirmed positive with Western blot. These 242 remaining were classified as newly diagnosed persons with HIV infection (1.56% sero-positivity). (Table 1)

**Table 1:** Rapid HIV testing outcomes and program costs in Emergency Department (Grady Memorial Hospital) and Jail (Fulton County Jail), Atlanta, GA.

	Emergency Department	Jail
**Testing outcome:**		
Testing program implementation period^a^	40 months	10.5 months
	(6/2008–9/2011)	(1/2011–12/2011)
Persons eligible for testing, N^b^	360,000	30,799
Persons offered HIV test, N	19,388	18,183
Persons received rapid HIV test, N (males; females; transgender)	15,510	11,819
	(7,562; 7,946; 2)	(9,467; 2,352; 0)
Proportion of eligible persons tested, %	4.31	38.37
Test acceptance rate of those offered a test, %	80.00	65.00
Rapid test preliminary positive, N (%)	268 (1.73)	130 (1.10)
New HIV diagnoses, N (%)^c^	242 (1.56)	41 (0.35)
**Program cost, $:**	721,363	274,226
Rapid test kit and material ($12.50/test kit)	193,875	147,738
Test kits and materials for running controls	8,970	8,000
Confirmatory tingtes with Western blot	10,300	5,356
Labor cost of counseling, testing, program administration^d^	508,218	113,132
Cost per person tested	46.51	23.20
Cost per preliminary HIV positive result	2,691.65	2,109.43
Cost per new HIV diagnosis	2,980.84	6,688.43

^a^ Number of months for the jail program excludes a 6 week period in mid-Summer 2011 in Fulton County Jail, when the contractor providing medical services changed.

^b^ The 30,799 admissions to the jail represented 29,392 unique persons, since some detainees were admitted more than once.

^c^In the Emergency Department, the new HIV diagnoses were not systematically validated with surveillance data housed in the health department.

^d^ In jail, nurse's time spent in counseling and testing was obtained through a time-motion study, average time per test 9.67 (S.D. 4.87) minutes; nurse's wage rate with fringe benefits was $20/hour; program administration time was based on annual salary with fringe benefits ($75,000). In the Emergency Department, approximately one-quarter of the labor cost represented administrative cost; the remaining three-quarters of the effort represented labor for counseling and testing.

During the 11-month study period at FCJ, 30,799 detainees were provided an intake medical evaluation and thus were eligible for HIV screening. While delays in training new staff, staffing shortages, and unavailability of test kits occurred periodically, 18,183 FCJ entrants actually had the opportunity to undergo HIV screening. HIV screening occurred on 11,819 (65.0%) detainees who were approached (9,467 men and 2,352 women); the remaining 35.0% declined an HIV test. One hundred and thirty tests were preliminarily positive and potentially represented new diagnoses. However, of the 130 persons with positive oral fluid test results, 89 were reclassified as previously diagnosed and 41 (31.5% of preliminary positive entrants) were confirmed as newly diagnosed cases of HIV infection (0.35% sero-positivity). (Table 1)

For the purpose of cost analyses, comprehensive program costs were used to calculate cost per patient and per newly diagnosed case of HIV infection. Costs of test kits ($12.50 per kit) were $193,875 (includes cost of donated kits) in the GMHED and $147,737 in the FCJ. The costs of running controls were $8,970 in the GMHED and $8,000 in the FCJ. Confirmatory Western blots were performed at total costs of $10,300 and $5,356 in the GMHED and the FCJ respectively. In terms of labor, for the GMHED testing program, the total labor cost for the 40-month period was $508,218, of which approximately $381,000 represented the labor of counseling and testing. Overall personnel cost for the FCJ testing program was $113,132 for the 46 weeks of testing, and included salary and fringe benefits for nurses ($38,132, calculated at $20/hour for work force primarily composed of licensed practical nurses) and an HIV educator ($75,000). On average, nurses spent 9.67 (S.D. 4.87) minutes per each test performed; during the time tests were developing, nurses could perform other duties, such as recording vital signs, and obtaining the prior medical history of a detainee-patient, thus test processing time was excluded in our cost analysis.

The GMHED’s total program cost was $721,363 with an average cost per test of $46.51. In the FCJ, the total cost of the program was $274,225, and the mean cost for each test was $23.20. The mean cost per new HIV diagnosis was $2,981 in the GMHED and $6,688 in the FCJ.

## Discussion

The HIV screening programs implemented in both the GMHED and FCJ were able to identify HIV-positive persons who were previously unaware of their infection. A recent review showed that cost per new diagnosis in screening programs ranged from $2,000 to $30,000.[24] The costs per new diagnosis in both of these settings are lower than comparable HIV testing cost analyses performed elsewhere. The cost per new HIV diagnosis may vary in these screening programs by the differences in methods for confirming a new HIV diagnosis, previously undiagnosed HIV infection, and the methods used to determine program costs. The GMHED cost for each newly identified HIV-positive diagnosis was $2,981 versus a median nationwide of $20,144 (2009 US$) for costs analyzed by program budget methods.[22] The jail’s cost of $6,688 per confirmed new HIV positive test was also lower compared to the median, nationwide figure of $15,018 for costs derived by microcosting-staff allocation methods. [22]

While neither program was able to offer screening to all potential patients, FCJ's integrated testing model utilizing jail nursing staff permitted broader testing coverage and resulted in lower costs per test. The GHMED’s parallel staffing model resulted in limited HIV screening due to lack of 24-hour availability of HIV test counselors. However, the high HIV test acceptance rate in the ED was encouraging. Implementation of an integrated HIV screening model in the GMHED would increase the availability of HIV testing services and likely increase the number of newly diagnosed cases of HIV infection.

Testing in each of these venues reaches patients who are at high risk of infection. Blacks are the population group most affected by HIV in the United States. Despite representing only 12% of the US population, in 2010, black men and women accounted for 44% of all new HIV infections for persons 13 years or older. Specifically, black men accounted for 70% of these new infections and 72% of those men identified as gay or bisexual, or other men who have sex with men (MSM).[25]

Previously published analyses from the GMHED testing program demonstrated that a vast majority of persons testing positive were black (95.2%) and male (75.2%),[19] and a disproportionate number of persons testing positive without a prior positive test in the GMH system were MSM (16.1%) compared to heterosexual male patients (1.5%).[18] Analysis of the FCJ data has also shown that 38% of men newly diagnosed with HIV are black men who have sex with men.[20] Given that nearly half (48.5%) of all health care provided to young black men is delivered through emergency departments [26] and one in five black men are incarcerated by age 34 years,[27] HIV screening in both emergency departments and jail sites in high HIV prevalence communities represents important opportunities to reach populations disproportionately affected by HIV in an efficient manner.

### Limitations

Ascertaining that a person had a newly reported HIV diagnosis was different for the two programs. The GMHED project was not able to systematically verify whether cases had previously been reported to the local or state HIV surveillance systems. This may have led to an overestimation of newly diagnosed HIV cases in the GMHED.

Labor of the jail program was integrated into the general medical care program at the facility. In the GMHED project, the labor of all program staff members who spent time in the HIV screening program was included in the cost estimates. In the jail project, individuals who solely worked on evaluation activities were not included in labor estimates. This difference in accounting may have led to an overestimation of costs for the GMHED project relative to the jail project.

As advancements in HIV diagnostic technologies improve the test sensitivity, shorten testing turnaround times, and reduce costs, EDs and large jails in communities with a high prevalence of HIV infection may consider applying new strategies and materials for implementing HIV screening. This may result in lower program costs and an increase in newly diagnosed cases of HIV infection in these already valuable screening venues.

## Summary

HIV screening in both the emergency department and jail venues yielded a high number of HIV diagnoses at low cost per new diagnosis. Different methods for confirming a new HIV diagnosis, previously undiagnosed HIV sero-positivity, and determination of program costs, prevent conclusions from being drawn that one location or testing program is more economical than the other.
